# The *Solo* Play of *TERT* Promoter Mutations

**DOI:** 10.3390/cells9030749

**Published:** 2020-03-19

**Authors:** François Hafezi, Danielle Perez Bercoff

**Affiliations:** Department of Infection and Immunity, Luxembourg Institute of Health, 29, rue Henri Koch, L-4354 Esch-sur-Alzette, Luxembourg; francois.hafezi@lih.lu

**Keywords:** *TERT* promoter mutations, telomerase, cell immortalization, GBM/glioma, melanoma, thyroid cancer, APOBEC mutations, UV mutations

## Abstract

The reactivation of telomerase reverse transcriptase (TERT) protein is the principal mechanism of telomere maintenance in cancer cells. Mutations in the *TERT* promoter (*TERTp*) are a common mechanism of TERT reactivation in many solid cancers, particularly those originating from slow-replicating tissues. They are associated with increased TERT levels, telomere stabilization, and cell immortalization and proliferation. Much effort has been invested in recent years in characterizing their prevalence in different cancers and their potential as biomarkers for tumor stratification, as well as assessing their molecular mechanism of action, but much remains to be understood. Notably, they appear late in cell transformation and are mutually exclusive with each other as well as with other telomere maintenance mechanisms, indicative of overlapping selective advantages and of a strict regulation of TERT expression levels. In this review, we summarized the latest literature on the role and prevalence of *TERTp* mutations across different cancer types, highlighting their biased distribution. We then discussed the need to maintain TERT levels at sufficient levels to immortalize cells and promote proliferation while remaining within cell sustainability levels. A better understanding of *TERT* regulation is crucial when considering its use as a possible target in antitumor strategies.

## 1. Introduction

Telomeres and their associated shelterin complex are located at chromosomal ends. Telomeres are tandem repeats of TTAGGG up to 15 kb long in humans. Together, telomeres and the shelterin complex protect chromosomal ends and preserve genomic DNA integrity [1,2,3,4]. Telomeres are shortened with each cell division. When telomere length falls below a critical threshold, cells become replicatively senescent and undergo apoptosis [5]. Cancer cells circumvent replicative telomere shortening by stabilizing them [6] through one of two mechanisms: reactivation of telomerase, the enzyme that extends telomeres (85–90% of cancers) [7,8,9,10], or homologous recombination between sister chromatids, a phenomenon termed alternative lengthening of telomeres (ALT) (3–10% of cancers) [10,11,12]. Telomerase is a ribonuclear holoenzyme composed of an RNA template (TERC) and a reverse transcriptase catalytic subunit (TERT) [1,2,3,4,13]. TERT is silent in most somatic cells, and is reactivated in cancer cells, endowing them with unrestricted proliferation capacity [6,14,15,16].

**Figure 1:**
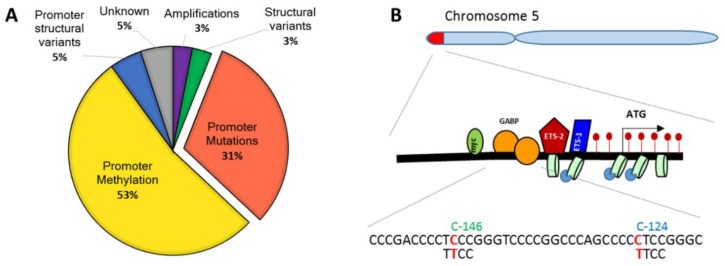
Mechanisms of telomerase reverse transcriptase (*TERT*) reactivation in cancer and *TERT* promoter (*TERTp*) mutations. (**A**) Different mechanisms of *TERT* reactivation in cancer according to Reference [10]. (**B**) Localization of *TERTp* mutations on Chromosome 5.

Although TERT activity is regulated principally at the transcriptional level (reviewed in References [3,4,9,17,18,19,20,21,22]), it may also be regulated through splicing [23,24], post-translational modifications, or intracellular trafficking [25,26,27,28]. The *TERT* promoter (*TERTp*) contains binding sites for numerous transcriptional activators including Sp-1, c-Myc, Hypoxia Induced Factor (HIF), AP-2, β-catenin, NF-κB, E-twenty-six (Ets)/ternary complex factors (TCF) family members, and transcriptional repressors (Wilms’ tumor (WT1), TP53, Nuclear Transcription Factor, X-Box Binding (NFX-1), Mad-1 and CCCTC binding factor (CTCF)) [3,4,9,17,18,19,20,21,29]. *TERT* expression can be reactivated by copy number variants (CNV), *TERT* or *TERTp* structural variants, chromosomal rearrangements juxtaposing *TERTp* to enhancer elements, cellular and viral oncogenes such as Hepatitis B virus (HBV) X protein (HBx) or high-risk Human papillomavirus (HPV)16 and HPV18 E6 oncoprotein, and, last but not least, mutations within *TERTp* (31% of TERT-expressing cancers) (Figure 1A) [10,30,31,32,33,34,35,36,37,38] (reviewed in [3,4,9,18,19,20,39]). Increased *TERTp* methylation is typically recorded in >50% of TERT-expressing tumors and cell lines [10,40,41,42,43,44,45,46,47]. Epigenetic regulation of *TERTp* is based on altered methylation patterns of specific regions. Hypomethylation of the region between −200 and −100 from the Translational Start site (TSS), encompassing the core promoter, enables binding of c-Myc and Sp-1, thus reactivating transcription. In contrast, the region spanning exon 1 (positions +1 to ±100 from the TSS) contains a binding site for the DNA insulator CTCF. Hypermethylation of this region disrupts binding of CTCF and therefore allows *TERT* transcription [41,42,43,44]. Similarly, the region between −600 and −200 from the TSS contains a second CTCF binding site and is partially hypermethylated in TERT-expressing cells [41,42,43,44]. The transcriptional control of *TERT* has been comprehensively reviewed recently [3,4,9,18,19,20,21,22,29,48] and, as such, is beyond the scope of this review. In this review, we focused on the distribution and exclusiveness of *TERTp* mutations.

## 2. Telomerase Reverse Transcriptase Promoter (*TERTp*) Mutations

*TERTp* mutations were first described in congenital and sporadic melanoma in 2013 [49,50]. Subsequent large-scale cohort studies together with seminal mechanistic studies both ascertained the *TERTp* mutation prevalence in many other forms of cancer and characterized their mode of action.

The two main *TERTp* mutations are located at positions 1,295,228 and 1,295,250 on Chromosome 5, and generate C to T transitions. They are located 124 and 146 base pairs upstream from the *TERTp* TSS (Figure 1B). Less frequent tandem mutations −125/−124 CC>TT and −139/−138 CC>TT have been reported in cutaneous tumors (Table 1) [49,51]. While these are somatic mutations, a germline mutation at position −57A>C from the TSS has been identified in familial melanomas and showed similar effects [49]. All of these mutations have similar effects, increasing TERT expression ~2–6 fold as measured through qRT-PCR, immunohistochemistry, TRAP, or reporter vectors in numerous cancer types, as outlined in Table 1 [37,50,52,53,54,55,56,57,58,59,60,61,62,63,64,65]. This increased TERT expression maintains self-renewal potential and telomeres in both stem cells and terminally differentiated bladder cells, indicating that these mutations are sufficient to immortalize cells [66,67].

All of these *TERTp* mutations (at positions −146, −124, −57, and −139/−138) create novel Ets/TCF transcription factor binding sites. The Ets/TCF transcription factors bind to GGAA motifs (or TTCC on the opposite strand). The 30 members of the Ets/TCF-family transcription factors are important contributors to oncogenesis and include Ets-1, Ets-2, and GA binding protein (GABP) [68]. So far, GABP has been reported to selectively bind the −124 C>T and −146 C>T mutations in GBM, melanoma, and urothelial bladder cancer cell lines [69,70,71]. Unlike the other Ets/TCF family transcription factors, GABP is an obligate dimer of GABPA and GABPB dimers. It binds two nearby in-phase GGAA sites [68,72,73,74] positioned 1, 2, or *n* helical turns away from each other [69], or brought close together by DNA looping [70]. *TERTp* mutations are associated with epigenetically active chromatin [54,69,75,76]. Intriguingly, whereas methylation of wild-type (wt) *TERT* promoter is associated with TERT expression [10,43,44], *TERTp* mutations are associated with decreased *TERTp* methylation [76]. The −146 C>T mutation was also shown to bind the non-canonical NF-κB-p52 and Ets-1/2 [59].

*TERTp* mutations have been recorded in a wide range of solid cancers. They are present in primary gliomas and glioblastoma multiforme (GBM), oligodendrogliomas and astrocytomas [10,40,52,53,54,57,58,60,64,65,77,78,79,80,81,82,83,84,85,86], melanomas, cutaneous basal cell carcinoma (BCC) and squamous cell carcinoma (SCC) [49,50,51,52,55,87,88,89,90,91], myxoid liposarcomas [77], urothelial bladder carcinoma [50,57,78,92,93,94], hepatocellular carcinoma (HCC) [50,57,62,95,96,97], and thyroid cancers [98,99,100,101,102,103,104,105,106], as well as oral and cervical SCC [36,37,57] (Table 1). Furthermore, they were consistently found in tumor cell lines derived from these malignancies [37,50,52,54,58,62,97,100,107,108]. *TERTp* mutations often arise in tissues with low rates of self-renewal (brain, thyroid) [77], where they provide an immediate competitive advantage to cells that acquire them. Conversely, they appear to be infrequent (<15%) in hematopoietic, lymphoid, or gastrointestinal malignancies. These are from compartments with high cellular turnover and intrinsic telomerase activity. Here, the endogenously elevated TERT levels likely render *TERTp* mutations neutral [3,38,57,77,109].

## 3. Cancer Distribution of *TERTp* Mutations

The clinicopathological association of *TERTp* mutations is cancer-dependent. It is a consideration for fine tumor stratification and orientation of patients towards personalized treatments, and provides insight into the process of cellular transformation.

### 3.1. Gliomas and Glioblastoma (GBM)

GBM are WHO Grade IV tumors of the central nervous system (CNS). Primary GBM evolve rapidly without prior low-grade lesions, while secondary GBM progress slowly from diffuse or anaplastic astrocytoma and oligodendroglioma (WHO Grade II and III). Primary and secondary GBM differ genetically more than histologically. The 2016 WHO classification of CNS tumors is based on “integrated diagnosis” including histology and isocitrate dehydrogenase (*IDH*)-*1/2* mutations (a biomarker for secondary GBM), and the presence of the 1p/19q codeletion (a marker for oligodendroglioma) [110]. *TERTp* mutations are relatively rare in diffuse (17.7%, range 10–19%) and anaplastic astrocytomas (24.7%, range 10–62.5%), as well as in *IDH*-mutated gliomas and secondary GBM (~28%). Their prevalence is highest (64.7%, range 45–88.6%) in oligodendrogliomas (where they coexist with the 1p/19q full deletion [53]) and in primary GBM (68%, range 44–100%) (Table 1) [38,52,53,65,77,80,81,84,85,111]. They tend to be found mainly in samples with epidermal growth factor receptor (*EGFR*) amplification, an early feature of primary GBM, [64,77,111]. Conversely, they appear to be mutually exclusive with mutations in α-thalassemia/mental retardation syndrome X-linked (*ATRX*) and Death Domain Associated Protein (*DAXX*) [38,65,77,79,80,110,111,112], two telomere-binding proteins mutated in ALT [11,12].

*TERTp* mutations are independently associated with older age, late clinical stage, poor prognosis, and shorter overall survival (OS) in GBM/glioma and *IDH*-wt astrocytoma patients. The presence of *TERTp* mutations alone is associated with a worse prognosis than *TERTp* mutations together with *IDH*-mutations [4,60,64,65,77,79,80,81,84,85,112]. Conversely, GBM patients with ALT and no *TERTp* mutations have longer OS than patients with *TERTp* mutations only [77,112,113]. In terms of treatment, Grade II and III *IDH*-wt CNS tumors generally respond to adjuvant radiation and chemotherapy with temozolomide (TMZ). However, the presence of *TERTp* mutations decreases sensitivity to genotoxic therapies. It has therefore been proposed to use *TERTp* mutations to further stratify *IDH*-wt Grade II and III gliomas into subgroups to orient treatment [60,81,114].

### 3.2. Melanoma and Non-Melanoma Skin Carcinoma

In patients with primary melanoma, *TERTp* mutations have been reported in 39.2% (range 22–71%) of tumors. They arise progressively in sun-exposed sites and have been attributed to UV radiation. They are associated with increased patient age, distal metastases, poor outcome, and compromised OS and disease-free survival (DFS) [49,50,51,52,88,89,115]. In ~50% of cases, they are associated with mutations in *BRAF*/*NRAS* [49,52,88,89,91,116], influencing OS in the following order: *TERTp^mut^*+*BRAF*/*NRAS^mut^*<*TERTp^mut^*~*BRAF*/*NRAS^mut^*<*TERTp*-wt+*BRAF*/*NRAS*-wt [56].

Consistent with their UV-induced origin in skin cancers, *TERTp* mutations are also highly prevalent at sun-exposed sites in non-melanoma squamous cell (50%) and basal cell carcinomas (46.2%, range 38–74%), the most common skin tumor [55,89,90]. *TERTp* mutations display unique features in melanoma and non-melanoma cancers. First, −146 C>T and −124 C>T occur with similar frequencies in contrast to all other cancers, where −124 C>T is by far the most prevalent mutation (Table 1). Second, −139/−138 CC>TT and −125/−124 CC>TT tandem mutations are often reported. Third, *TERTp* mutations were detected in 9/10 melanomas with ALT in one study [117] and together (−124 C>T + −146 C>T) in two patients with BCC in another study [89], indicating that more than one telomere maintenance mechanism can, unusually, coexist in skin cancers.

### 3.3. Urothelial Bladder Cancer

*TERTp* mutations have been detected in 64.6% (range 29.5–100%) of urothelial bladder and upper urinary tract cancers. They are the most common somatic lesions in this cancer type [52,57,61,77,92,94,118,119]. They have been associated with reduced survival, disease recurrence, and distal metastases [61,118,119], although there appears to be no difference between early- and late-stage patients [52,94].

### 3.4. Thyroid

Among thyroid cancers, *TERTp* mutations have been reported mainly in follicular-cell-derived thyroid malignancies (papillary thyroid carcinoma (PTC): 13.4%, range 4.1–37.7%; follicular thyroid carcinoma (FTC): 13.9%, range 5.9–66.7%; poorly differentiated thyroid carcinoma (PDTC): 43.7%, range 21–51.7%; and anaplastic thyroid carcinomas (ATC): 39.7%, range 13–50%). The presence of *TERTp* mutations is significantly associated with increased age, tumor size and stage, distal metastases, tumor recurrence, and shorter OS and DFS in PTC and FTC. Their prevalence increases from differentiated PTC and FTC to the more aggressive poorly differentiated ATC (Table 1) [98,99,100,101,102,103,104,105,106]. The association of TERTp mutations with the common *BRAF*-V600E mutation is a powerful predictor of poor OS and DFS [52,98,99,104,105,106,108]. As in glioma, *TERTp* mutations compromise the outcome of radioiodine therapy [101,105].

### 3.5. Hepatocellular Carcinoma (HCC)

*TERTp* mutations are an early event in hepatocellular tumorigenesis [57,62,77,95]. They are not only seen in established HCC (47.1%, range 29.3–65.4%). As hepatocellular adenomas transform into HCC, *TERTp* mutations are the first gene recurrently mutated after β-catenin (*CTNNB1*) in preneoplastic cirrhotic lesions [62,95]. Together with the *CTNNB1* mutation, *TERTp* mutations are considered critical effectors of malignant transformation. As such, they have been proposed as early biomarkers for hepatocellular transformation [62,77,95,96,120,121].

*TERTp* mutations appear to be more frequent in HCV-associated HCC [62,77,95,96,122] and less frequent or excluded from HBV-associated HCC [62,96,121,122], although this remains controversial [63,77,95]. HBV DNA insertion in the *TERTp* is a recurrent mechanism of TERT transcriptional reactivation in HBV-associated HCC [34,123,124], and a genetic screen of *TERT* in HCC found *TERTp* mutations to be mutually exclusive with HBV integration, *TERT* CNVs, and *ATRX* mutations [121].

### 3.6. Cervical and Oral Head and Neck Squamous Cell Carcinoma (HNSCC)

*TERTp* mutations were detected in cervical SCC (20.1%, range 4.5–21.8%) and HNSCC (22.5%, range 17–31.7%) [36,37]. These malignancies are often associated with high-risk-HPV16/18 E6 and E7 viral oncoproteins and with APOBEC mutations [125,126,127]. High-risk HPV–E6 transactivates *TERT* [30,32,33,128,129]. *TERTp* mutations have a notably higher prevalence in HPV-negative cervical and oral SCC. This gives distinct patterns of *TERT* reactivation through mutually exclusive mechanisms [36,37]. In cervical SCC, they are associated with higher TERT levels than HPV16/18-E6-positive tumors and with advanced cervical cancer [36,37]. Broader studies are needed to evaluate the added value of screening for the molecular mechanism underlying TERT reactivation in cervical and oral SCC.

### 3.7. The rs2853669 Polymorphism

Among *TERT* polymorphisms, a common polymorphism (rs2853669 A>G) which disrupts a pre-existing Ets/TCF binding site located 245 bp upstream of the *TERT* TSS has been reported to modify the effect of *TERTp* mutations. It decreases *TERT* transcription in vitro and reverses *TERT* upregulation by *TERTp* mutations [56,61,81,85,130]. Controversial clinical impacts have been reported, from a beneficial effect on OS and limited tumor recurrence in *TERTp*-mutated urothelial bladder cancer, renal clear cell carcinoma, melanoma, and GBM [56,61,81,85,116,131], to unchanged or worsened clinical outcome in GBM, melanoma, or differentiated thyroid carcinomas [64,65,84,91,102,103]. In HCC, the rs2853669 polymorphism in combination with *TERTp* mutations has been associated with decreased OS and DSF, and increased *TERTp* methylation and expression [47]. Possible reasons for these conflicting reports could be homozygosity versus heterozygosity of the variant, or its occurrence on the same allele as *TERTp* mutations. Further studies are needed to assess the relevance of screening for this polymorphism for prognostic and treatment purposes.

## 4. Cancer Bias of *TERTp* Mutations

**Figure 2:**
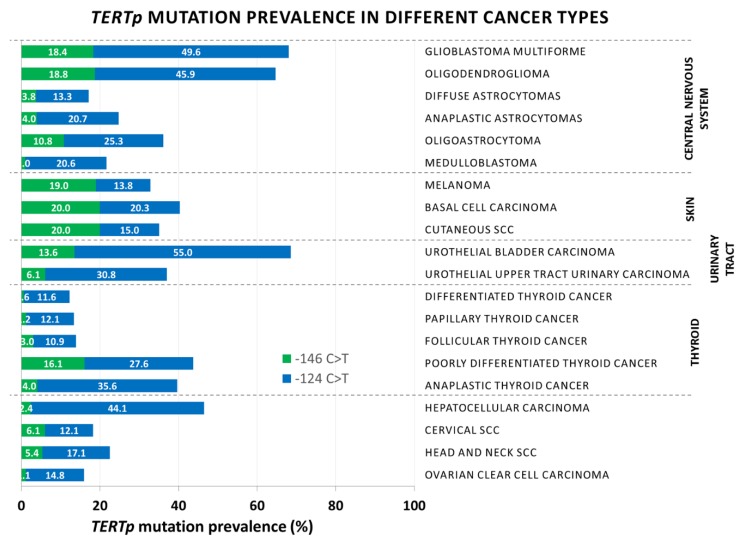
Distribution of *TERT* promoter mutations in different cancers.

*TERTp* mutations have been recorded in individuals of Caucasian, African, and Asian descent, with no race-related bias. The −124 C>T mutation has an overwhelmingly higher prevalence than the −146 C>T mutation in all cancers, with the exception of skin cancers, where both hotspots are mutated with comparable frequencies (Figure 2 and Table 1). Although both −124C>T and −146C>T mutations generate identical sequences, enable binding of GABPA, and are equally efficient in increasing *TERT* transcription in vitro [57,69], in vivo, the −124 C>T mutation was associated with higher TERT mRNA in GBM [57,112]. This would suggest that the Ets/TCF binding site at position −124 provides a more favorable or accessible hotspot for the transcriptional machinery [109]. The overrepresentation of the −146 C>T mutation in skin cancers hints at different etiologies of *TERTp* mutations. *TERTp* mutations in melanoma and non-melanoma skin cancers have been attributed to UV damage [49,51,55,88,89,90,91,116], which triggers C→T transitions at CC dinucleotides [55,127]. Nevertheless, C→T transitions where C is preceded by C also conform to the preferred target of Apolipoprotein B mRNA Editing Catalytic Polypeptide-like (APOBEC)3A/B de-aminations and to aging mutations [127,133]. APOBEC3 mutations are highly prevalent in ovarian and HPV-associated cervical and oral SCC [125,126,127], as well as in HCC and in cirrhotic lesions [121,134]. A role for APOBEC and aging-associated de-aminations is consistent with potentially increased accessibility of the −124 position to DNA binding proteins and with the association of *TERTp* mutations with older age at diagnosis in GBM, melanoma, and PTC [52,57,60,63,64,77,79,80,82,86,88,98,100,101,102]. These observations therefore raise the possibility that UV-driven lesions account for *TERTp* mutations in skin cancers, while APOBEC and age-driven de-aminations account for the −124 C>T mutation in other cancers. Further epidemiological and mechanistic studies are needed to shed light on this point.

The −139/−138 CC>TT tandem mutation is very infrequent, limited to skin cancers, and has been associated with lower DFS. This tandem mutation has been suggested to favor chromosomal instability [51].

## 5. Exclusiveness of *TERTp* Mutations

Aside from non-melanoma skin cancers [90], *TERTp* mutations are mostly monoallelic. This suggests that TERT reactivation on one allele is probably sufficient to ensure telomere maintenance or elongation in cancer cells [54]. In line with this observation, *TERTp* mutations appear to be mutually exclusive [50]. Likewise, *TERTp* mutations are generally absent from cancers where telomere elongation is ensured by ALT [77,79,80,98] or *TERT* copy-number duplications [38,121]. *TERTp* mutations are also less frequent in cancers where viral transformation or viral oncogenes reactivate *TERT* transcription, such as HBV-DNA or high-risk HPV16/18 E6 [30,32,33,36,37,62,95,96,121,122]. These observations reinforce the concept that, despite some exceptions [38,89,111,117], tumors generally rely on one mechanism for telomere maintenance. The reasons for such selectivity remain speculative to date. One possible explanation is that there is a threshold for TERT expression, above which the biological advantage is lost.

Consistent with this view, Phosphatidyl Inositol Kinase 3 (PIK3) CA and PIK3 Receptor 1 (PIK3R1) mutations are recorded in 50% of GBM with wt *TERTp* and tend to be mutually exclusive with *TERTp* mutations in ovarian clear cell carcinoma [79,86,132]. The PIK3CA/Akt signaling pathway is involved in cellular self-renewal in embryonic stem cells and cancer stem cells [135], as well as in TERT Ser227 and Ser824 phosphorylation, subsequent nuclear translocation, and cellular transformation [25,26,27,28]. Mutual exclusion of *PIK3CA* and *TERTp* mutations suggests that activation of the PIK3CA/Akt pathway or of TERT confer cells a similar growth and proliferative advantage. In the absence of TERT reactivation, other telomere maintenance mechanisms, such as ALT, can achieve immortalization [27]. Indeed, TERT also contributes to cell survival and proliferation through telomere-independent mechanisms; it facilitates Wnt/β-catenin-dependent [136,137], c-myc-dependent [138,139], and NF-κB-dependent gene transcription [140,141], thereby sustaining both oncogenic signaling pathways and its own transcription in a feedforward loop [29,142]. It also regulates methylation [48,143] and DNA damage responses [144,145], and protects cells from Endoplasmic Reticulum (ER) stress and apoptosis by buffering Reactive Oxygen Species (ROS) and modulating mitochondrial function [145,146,147,148,149,150,151]. It is highly likely that TERT homeostasis is also tuned by these functions within a given tumor type and microenvironment, and by related metabolic alterations that need to be preserved.

## 6. Discussion

Hints for a model come from the observation that overall, *TERTp* mutations are associated with late-stage disease in GBM, melanoma, urothelial, and thyroid carcinoma [49,52,60,61,66,85,98,100,101,103,104,105,112,118] and with the last steps of hepatocellular transformation [62,95]. They often occur with or after mutations in pathways associated with cell growth and proliferation. In GBM, *TERTp* mutations coexist with *EGFR* amplification [64,77,111], and in urothelial bladder carcinoma, they are associated with *FGFR3* (Fibroblast Growth Factor Receptor 3) mutations [61,94]. In ~50% of melanoma, urothelial, and thyroid cancers, *TERTp* mutations coexist with the common *BRAF*-V600E mutation [52,88,89,105,106,108,116,152]. GFR and BRAF/RAS kinases control the MAPK and PI3K-Akt pathways that lead to cell growth, survival, and angiogenesis. Constitutive activation of the GFR/FGFR-BRAF/RAS pathway leads to constitutive cell growth and division [153]. Mutations in these oncogenes are often detectable in low-grade tumors and probably precede *TERTp* mutations [22,61,77,112]. The picture is even more clear-cut in HCC, where mutations in β-catenin (*CTNNB1*) neatly precede *TERTp* mutations during the process of malignant transformation [62,95,120]. β-catenin is involved in cell adhesion and interacts with Wnt, promoting cell growth and division. The proliferative advantage conferred by driver mutations in these pathways leads to accelerated telomere erosion. Accordingly, most tumors display telomere dysfunction and shortened telomeres, which leads to chromosome instability [10,22,61,66,98,112,115]. In this scenario, TERT reactivation regenerates telomeres sufficiently to maintain them above the critical threshold and to stabilize the tumor genome [3,18,145]. This interpretation is consistent with the association of *TERTp* mutations with shortened telomeres and with age as in PTC, melanoma, and GBM/glioma, since cells from younger patients or with sufficiently long telomeres do not need to rely on telomerase reactivation to overcome telomeric crisis [10,29,57,77,85,98,101,115]. Partial telomere healing is coherent with a modest increase in TERT expression (2- to 4-fold) and with a single genetic mechanism of telomere elongation. It likely reflects an exquisite balance between escape from apoptosis resulting from telomere attrition and genomic instability, and cell sustainability in terms of oxygen and nutrient supplies.

Intriguingly, it was recently reported that GABPA controls the cell cycle and induces cell differentiation, thus acting as a tumor suppressor regulating cell proliferation, stemness, and adhesion. It decreased tumor invasiveness and distal metastases in PTC, HCC, and bladder carcinoma [154,155,156]. GABPA levels were decreased and even negatively associated with TERT expression in PTC [154,155,156]. One possible explanation is that other Ets/TCF family transcription factors bind *TERTp* mutations. Alternatively, the decrease in GABPA expression may follow rather than precede *TERTp* mutations. In this case, it would be a cellular adaptation which confers a selective advantage to *TERTp*-mutated (and *GFR*/*BRAF*/*RAS*-mutated) cells by containing TERT reactivation within sustainable limits. Decreased GABPA could also be an adaptation to the TERT-induced proliferation, stemness, and invasion to avoid contradictory signals. Further studies establishing the order of emergence of these mutations would be needed to shed light on this matter.

Taken together, these observations point to a fine tuning of TERT homeostasis and suggest that there is a narrow kinetic and quantitative window for TERT expression. Below that window, cells succumb to telomere crisis and DNA damage. Above that window, cells succumb to overwhelming genetic alterations or metabolic needs. This frailty could be exploited through strategies aiming to push cells either way beyond the threshold of TERT tolerability.

## 7. Concluding Remarks

*TERTp* mutations have only been described recently; however, they have prompted an impressive number of studies which draw a comprehensive picture of their prevalence across cancers, as well as providing clues on their mechanisms of action and their associated constraints. They have been proposed as potential biomarkers with predictive and treatment-orienting value. However, more structured studies are needed to validate their clinical potential, particularly since they appear at different stages in different malignancies, ranging from preneoplastic cirrhotic lesions to late stage GBM or melanoma with distal metastases. Cancer cells only require one mechanism of telomere maintenance. This underscores the key role of telomere stabilization in the process of transformation, as well as the necessity of maintaining an exquisitely balanced TERT homeostasis to achieve tumor cell selection, adaptation, and sustainability. TERT is a target of choice in antitumor strategies due to its reactivation in numerous cancers. A better understanding of TERT regulation, homeostasis, and functions could help to overcome the shortcomings of prior genetic and immunotherapy-based approaches targeting TERT.

## Figures and Tables

**Table 1:** Prevalence and distribution of telomerase reverse transcriptase promoter (*TERTp*) mutations in cancer genomes. The prevalence of *TERTp* mutations is given as percentage and as total number of cases.

Cancer Type	Stage	Prevalence of Mutations	−146 C>T	−124 C>T	Tert Upregulation	Methods	Sample Origin	Remarks	Ref.
Central nervous system (CNS)									
GBM		62% 24/39	25% 6/24	75% 18/24	Yes	DNA sequencing, qRT-PCR, IHC,	Patients (Portugal)	Associated with older age.	[52]
GBM	IV	83.9% 47/55	34% 16/47	65.9% 31/47	Yes	DNA sequencing, qRT-PCR, TRAP, reporter assays	Patients (China)	Associated with older age.	[57]
GBM (Primary)	IV	83% 65/78	24.6% 16/65	75.4% 49/65	N/A	DNA sequencing	Patients (US American)	Associated with shorter OS, *IDH*-wt, *ATRX*-wt, exclusively in *EGFR^mut^* samples.	[77]
GBM	I–IV	44.6% 45/101	26.7% 12/45	73.3% 33/45	Yes	DNA sequencing, qRT-PCR, reporter assays	Patients (China)	Associated with late-stage disease and patient age. Only in gliomas, not in pituitary adenocarcinomas, meningiomas or secondary metastases.	[60]
GBM		55% 197/358	27% 54/197	73% 144/197	N/A	DNA sequencing	Patients (Switzerland)	Associated with shorter OS and with *EGFR^mut^*. Negatively associated with mutant *IDH* and *TP53*. More frequent in primary (58%) than in secondary GBM (28%). One patient with both −146 C>T + −124 C>T mutation.	[111]
GBM (primary & secondary)	IV	80.3% 143/178	*	*	N/A	DNA sequencing	Patients	Associated with shorter OS in patients without rs2853669 *TERT* -245 A>G polymorphism. Detected in 4/14 (28%) secondary GBM.	[81]
GBM	IV	66.9% 141/211	25.5% 36/141	74.5% 105/141	N/A	DNA sequencing	Patients (Portugal & Brazil)	Associated with older age, poor prognosis, and shorter survival. Reversed by rs2853669 *TERT* −245 A>G polymorphism.	[85]
GBM		60.4% 29/48	24.1% 7/29	75.8% 22/29	Yes	DNA sequencing, qRT-PCR	Patients (Korea)	Associated with older age. Not associated with OS or DFS. Associated with *MGMT* methylation and *EGFR* amplification. Associated with rs2853669 *TERT* −245 A>G polymorphism (21/29 patients). rs2853669 *TERT* −245 A>G polymorphism reversed TERT upregulation by *TERTp* mutations.	[64]
GBM		73% 92/126	28% 26/92	82% 66/92	Yes	DNA sequencing, qRT-PCR, TRAP, qPCR		Mutually exclusive with *IDH*-1 mutations. Associated with shorter telomeres. Associated with lower OS in *IDH*-1wt patients. rs2853669 *TERT* -245 A>G polymorphism associated with improved OS in patients without *TERTp* mutations, and with worse OS in patients with *TERTp* mutations.	[65]
GBM (primary)		86% 79/92	25% 20/79	75% 69/79		DNA sequencing		Associated with older age and shorter OS. Homozygous rs2853669 *TERT* −245 A>G polymorphism associated with worse OS in patients without and with *TERTp* mutations.	[84]
GBM and gliomas (primary)		100% 10/10	10% 1/10	90% 9/10	N/A	DNA sequencing	Patients	In primary GBM, characterized by 10q deletion *EFGR* amplification.	[58]
GBM		94% 33/35	36% 12/33	64% 21/33	2.2–286-fold compared to normal astrocytes	DNA sequencing, qRT-PCR	Cell lines		[58]
Total GBM		905/1331 (68%)	206/762 (27%)	567/762 (73%)					
Oligodendroglioma	II	45% 10/22	20% 2/10	80% 8/10	Yes	DNA sequencing, qRT-PCR, IHC	Patients (Portugal)		[52]
Oligodendroglioma	II–III	70% 7/10	14.3% 1/7	85.7% 6/7	Yes	DNA sequencing, qRT-PCR, TRAP, Reporter Assays	Patients (China)	Associated with older age.	[57]
Oligodendroglioma	II–III	46.3% 25/54	24% 6/25	76% 19/25	N/A	DNA sequencing	Patients (Portugal & Brazil)	Associated with older age at diagnosis. Not associated with lower survival.	[85]
Oligodendroglioma		73.5% 25/34	20% 5/25	80% 20/25	Yes	DNA sequencing, qRT-PCR	Patients (Japan)	Associated with total 1p19q loss and *IDH-1*/2 mutations (98%) but exclusive with *IDH*-*1^mut^* if not total loss of 1p19q.	[53]
Oligodendroglioma	II–IV	66.81% 151/226	*	*	N/A	DNA sequencing	Patients (US American)	Associated with shorter OS. Can be associated with *ATRX* mutations or *IDH^mut^*/1p19q loss.	[80]
Oligodendroglioma	II–III	63.2% 12/19	41.7% 5/12	58.3% 7/12	N/A	DNA sequencing	Patients (US American)	*IDH*-wt only. Associated with worse prognosis in *IDH*-wt. Associated with older age. Mutually exclusive with *ATRX* mutations.	[77]
Anaplastic oligodendroglioma	III	54% 13/24	30.8% 4/13	69.2% 9/13	Yes	DNA sequencing, qRT-PCR, IHC,	Patients (Portugal)	Associated with older age.	[52]
Anaplastic oligodendroglioma		74.2% 23/31	30.4% 7/23	69.6% 16/23	Yes	DNA sequencing, qRT-PCR	Patients (Japan)	Associated with total 1p19q loss and *IDH*-1/2 mutations (98%) but exclusive with *IDH*-1 if not total loss of 1p19q.	[53]
Anaplastic oligodendroglioma	III	88.5% 23/26	43.5% 10/23	56.5% 13/23	N/A	DNA sequencing	Patients (US American)	Associated with older age. *IDH*-wt only. Associated with worse prognosis in *IDH*-wt. Mutually exclusive with *ATRX* mutations.	[77]
Total Oligodendroglioma		289/446 (64.7%)	40/138 (29%)	98/138 (71%)					
Diffuse astrocytomas		19.2% 10/52	20% 2/10	80% 8/10	Yes	DNA sequencing, qRT-PCR	Patients (Japan)	Associated with total 1p19q loss and *IDH*-1/2 mutations (98%) but exclusive with *IDH*-1 if not total loss of 1p19q.	[53]
Diffuse astrocytoma	II	15% 3/20	33,3% 1/3	66,6% 2/3	Yes	DNA sequencing, qRT-PCR, IHC	Patients (Portugal)	Associated with older age.	[52]
Diffuse astrocytoma	II	20% 8/40	25% 2/8	62.5% 5/8	Yes	DNA sequencing, qRT-PCR, TRAP, reporter assays	Patients (China)	Associated with age.	[57]
Diffuse astrocytoma	II	15.2% 7/46	16.7% 1/7	83.3% 6/7	N/A	DNA sequencing	Patients (Portugal & Brazil)	Frequency increased with grade.	[85]
Total Diffuse Astrocytoma		28/158 (17.7%)	6/28 (21.4%)	21/28 (75%)					
Astocytoma	II–IV	62.5% 416/665	N/A	N/A	N/A	DNA sequencing	Patients (US American)	Associated with shorter OS. Can be associated with *ATRX* mutations or IDHmut/1p1q loss.	[80]
Anaplastic Astrocytomas	III	10% 1/10	0% 0/1	100% 1/1	N/A	DNA sequencing	Patients (Portugal & Brazil)	Frequency increased with grade.	[85]
Anaplastic Astrocytoma	III	33.3% 4/12	0% 0/4	100% 4/4	Yes	DNA sequencing, qRT-PCR, TRAP, reporter assays	Patients (China)	Correlation with age.	[57]
Anaplastic Astrocytomas	III	25.3% 20/79	20% 4/20	80% 16/20	Yes	DNA sequencing, qRT-PCR	Patients (Japan)	Associated with total 1p19q loss and *IDH*-1/2 mutations (98%) but exclusive with *IDH*-1 if not total loss of 1p19q.	[53]
Total Anaplastic Astrocytomas		25/101 (24.7%)	4/25 (16%)	21/25 (84%)					
Mixed Oligoastocytoma	II–IV	32.3% 63/195	*	*	N/A	DNA sequencing	Patients (US American)	Associated with shorter OS. Can be associated with *ATRX* mutations or *IDH^mut^*/1p1q loss.	[80]
Oligoastrocytoma		40% 14/35	28.6% 4/14	71.4% 10/14	Yes	DNA sequencing, qRT-PCR	Patients (Japan)	Associated with total 1p19q loss and *IDH*-1/2 mutations (98%) but exclusive with *IDH*-1 if not total loss of 1p19q.	[53]
Oligoastrocytoma	II–III	40.0% 4/10	50% 2/4	50% 2/4	N/A	DNA sequencing	Patients (Portugal & Brazil)	Not associated with lower survival.	[85]
Anaplastic Oligoastrocytoma		48.9% 22/45	27.3% 6/22	72.7% 16/22	Yes	DNA sequencing, qRT-PCR	Patients (Japan)	Associated with total 1p19q loss and *IDH*-1/2 mutations (98%) but exclusive with *IDH*-1 if not total loss of 1p19q.	[53]
Total Oligoastrocytoma		103/285 (36.1%)	12/40 (30%)	28/40 (70%)					
Medulloblastoma		33.3% 2/6	50% 1/2	50% 1/2	N/A	DNA sequencing	Patients (China)	Associated with age.	[57]
Medulloblastoma		20.9% 19/91	0%0/19	100% 19/19	N/A	DNA sequencing	Patients (US American)	*IDH*-wt and *ATRX*-wt only. Associated with worse prognosis in *IDH*-1-wt. Associated with older age. Mutually exclusive with ALT.	[77]
Total Medulloblastoma		21/97 (21.6%)	1/21 (4.7%)	20/21 (95.3%)					
Skin									
Melanoma		71% 50/70	46% 23/50	54% 27/50	Yes	DNA sequencing, reporter vectors	Patients & cell lines		[50]
Melanoma		32.5% 25/77	20% 5/25	28% 7/25	N/A	DNA sequencing	Patients	-57 C>T germline mutation in family with history of melanoma. High prevalence in metastatic cell lines (85%) compared to primary melanoma (32.5%). CC>TT −139/−138 tandem mutation in 10.4% patients. Concomitant with *BRAF* mutations in 47% of cases.	[49]
Melanoma		29% 16/56	50% 8/16	50% 8/16	N/A	DNA sequencing	Patients (Portugal)	Associated with *BRAF* mutations.	[52]
Melanoma		34% 97/287	52.5% 51/97	36% 35/97	Yes	DNA sequencing, qRT-PCR	Patients (Spain)	CC>TT −139/−138 tandem mutations in 4/97 (4.1%) patients. Associated with *BRAF* mutations in 50% cases.	[88]
Melanoma		41.6% 121/291	*	*	N/A	DNA sequencing	Patients (Spain)	Associated with shorter telomeres in tumor and with accelerated telomere shortening rate. Associated with *BRAF*/*NRAS* mutation in 75/243 cases. Telomere shortening rate: *BRAF*/NRAS^mut^+*TERTp^mut^*>*TERTp^mut^*>*BRAF*/*NRAS^mut^*	[115]
Melanoma		22% 26/116	35% 9/26	46% 12/26	Yes	DNA sequencing, IHC	Patients (Portugal)	Associated with reduced OS & DFS. More prevalent in sun-exposed regions. Associated with increased mitotic rates. −138/−138 CC>TT tandem mutation in 2/26 (7.7%) patients. −125/−124 CC>TT tandem mutation in 3/26 (11.5%) patients. Associated with *BRAF*-V600E mutation (58% of cases).	[89]
Melanoma		38.6% 116/300	50% 58/116	32.8% 32/116	N/A	DNA sequencing	Patients (Spain)	Associated with shorter OS and DFS. −139/−138 CC>TT & −125/−124 CC>TT tandem mutations in 16/116 cases (13.8%). Associated with *BRAF*/*NRAS* mutations in 126/283 (44.5%) cases. Reversed by rs2853669 *TERT* -245 A>G polymorphism.	[116]
Melanoma		54.8% 63/115	61.9% 39/63	30.2% 19/63	N/A	DNA sequencing	Patients (Austria)	−139/−138 CC>TT tandem mutations in 4/63 (6.3%) patients. −125/−124 CC>TT tandem mutation in 1/63 (1.6%) patient. Associated with *BRAF*/*NRAS* mutation in 75/243 cases. Associated with rs2853669 *TERT* -245 A>G polymorphism.	[91]
Total Melanoma		514/1312 (39.2%)	193/398 (48.5%)	140/398 (35.1%)					
Basal cell carcinoma		55.6% 18/32	55.6% 10/18	22.2% 4/18	N/A	DNA sequencing	Patients (Germany)		[55]
Basal cell carcinoma (sporadic & nevoid)		74% 31/42	35.5% 11/31	45.1% 14/31	N/A	DNA sequencing	Patients	Mostly homozygous. −139/−138 CC>TT tandem mutation in 7/31 (22.6%) patients. −125/−124 CC–TT tandem mutation in 5/31 (16.1%) patients. 1 patient with −139/−138 CC>TT + −125/−124 CC>TT tandem mutations. Mutations more frequent in basal cell carcinoma than in squamous cell carcinoma.	[90]
Basal cell carcinoma		38.7% 76/196	43% 33/76	49% 37/76	no	DNA sequencing, IHC	Patients (Portugal)	No correlation with clinical parameters. Higher prevalence in patients not exposed to X-irradiation: 48/94 (51%) vs. 28/102 (27%) in X-irradiated patients. −124 C>T more frequent than −146 C>T in non-X-irradiated patients; −146 C>T more frequent in X-irradiated patients. −139/138 CC>TT tandem mutation in 2/76 (2.6%) patients, 2 patients with −146 C>T + −124 C>T mutations.	[89]
Total Basal cell carcinoma		125/270 (46.2%)	54/125 (43.2%)	55/125 (44%)					
Cutaneous SCC		50% 17/34	29.4% 5/17	29.4% 5/17	N/A	DNA sequencing	Patients (Germany)		[55]
Cutaneous SCC		50% 13/26	54% 7/13	31% 4/13	N/A	DNA sequencing	Patients	Mostly homozygous. −139/−138 CC>TT tandem mutation in 2/13 (15.4) patients. Mutations more frequent in basal cell carcinoma than in squamous cell carcinoma.	[90]
Total Cutaneous SCC		30/60 (50%)	12/30 (40%)	9/30 (30%)					
Bladder/urinary tract cancers									
Bladder Cancer		85% 44/52	4.5% 2/44	95.5% 42/44	N/A	DNA sequencing	Patients (China)		[78]
Urothelial bladder carcinoma	III	80% 12/15	17% 2/12	83% 10/12	N/A	DNA sequencing	Patients (US American)		[93]
Urothelial bladder carcinoma		66.7% 14/21	28.6% 4/14	71.4% 10/14	N/A	DNA sequencing	Patients (US American)		[77]
Urothelial bladder carcinoma		61.7% 148/240	25% 37/148	58.8% 87/148	N/A	DNA sequencing	Patients (China)	Not associated with age.	[57]
Urothelial bladder carcinoma		59% 48/82	37.5% 18/48	62.5% 30/48	N/A	DNA sequencing, qRT-PCR	Patients (Portugal)	Not associated with age. Low-grade bladder cancer: 67%, high-grade bladder cancer: 56%.	[52]
Urothelial bladder carcinoma		65.4% 214/327	17.8% 38/214	81.8% 175/214	N/A	DNA sequencing, relative telomere length	Patients (Sweden)	Associated with shorter telomeres and worse OS. Associated with *FGFR3* mutation in 45% of tumors. *FGFR3* mutations found in low-grade tumors, *TERTp* mutations in low-grade and high-grade tumors. Reversed by rs2853669 *TERT* −245 A>G polymorphism.	[61]
Urothelial bladder carcinoma		77.1% 361/468	17% 62/361	83% 299/361	Not increased	DNA sequencing, qRT-PCR	Patients	Not associated with OS, DFS, or clinical outcome. Associated with *FGFR3^mut^*.	[94]
Urothelial bladder carcinoma		100% 33/33	12% 5/33	85% 28/33	N/A	DNA sequencing	Patients	Pure micropapillary carcinoma and urothelial cancer with focal micropapillary features.	[92]
Urothelial upper tract urinary carcinoma		76.9% 40/52	12.5% 5/40	72.5% 29/40	N/A	DNA sequencing	Patients (China)	Not associated with age.	[57]
Urothelial upper tract urinary carcinoma		47.4% 9/19	11.1% 1/9	88.9% 8/9	N/A	DNA sequencing	Patients (US American)		[77]
Urothelial upper tract urinary carcinoma		29.5% 65/220	18.5% 12/65	81.5% 53/65	N/A	DNA sequencing, Detection in urine	Patients (China)	Associated with distant metastases.	[118]
Total Urothelial bladder & upper tract urinary carcinoma		988/1529 (64.6%)	186/988 (18.8%)	771/988 (78%)					
Thyroid									
Differentiated thyroid cancer		12.2% 41/336	4.9% 2/41	95.1% 39/41	N/A	DNA sequencing	Patients	Only in malignant lesions.	[108]
Papillary thyroid cancer		8% 13/169	7.7% 1/13	84.6% 11/13	Yes	DNA sequencing, qRT-PCR, IHC	Patients (Portugal)		[52]
Papillary thyroid cancer	III/IV	11.3% 46/408	15.2% 7/46	85.8% 39/46	N/A	DNA sequencing	Patients (China)	Associated with older age, larger tumor size, extrathyroid invasion, advanced clinical stage. Associated with *BRAF*-V600E mutation.	[99]
Papillary thyroid cancer		27% 13/51	7.7% 1/13	92.3% 12/13	N/A	DNA sequencing	Patients (Sweden)	Only in patients >45. Correlated with shorter telomeres and distal metastases. PTC: 27% (25/332); FTC: 22% (12/70); ATC: 50% (12/36).	[98]
Papillary thyroid cancer	III/IV	4.1% 18/432	*	*	N/A	DNA sequencing	Patients (Korea)	Associated with *BRAF*/*RAS* mutations. Associated with tumor size, stage III-IV, recurrence, decreased OS and DFS with BRAF/RAS mutations: *RAS*/*BRAF* >*TERTp* > R*A*S/*BRAF*+*TERTp*.	[106]
Papillary thyroid cancer		11.7% 30/257	0% 0/30	100% 30/30	N/A	DNA sequencing	Patients	Only in malignant lesions. −124 C>T associated with *BRAF*-V600E mutation.	[108]
Papillary thyroid cancer		37.7% 10/27	10% 1/10	90% 9/10	N/A	DNA sequencing	Patients (Korea)	No *TERTp* mutation found in 192 well differentiated cancers without distant metastasis.	[105]
Papillary thyroid cancer		22% 18/80	44% 8/18	66% 10/18	N/A	DNA sequencing	Patients (US & Japan)	More frequent in *BRAF*-wt patients than in *BRAF^mut^*.	[100]
Papillary thyroid cancer		31.8% 77/242	0% 0/77	100% 77/77	N/A	DNA sequencing	Patients (US)	Associated with older age (>45 years), larger tumor size, stage III–IV, distant metastases, decreased OS and DFS. rs2853669 *TERT* −245 A>G polymorphism (46.7% (113/242)of patients) increases OS & DFS in patients without *TERTp* mutations and with *BRAF*-V600E.	[103]
Papillary thyroid cancer		12% 22/182	14.6% 3/22	86.4% 19/22	Yes	DNA sequencing, WB, and IHC	Patients (Italy)	Associated with older age and poor prognosis. Increased cytoplasmic localization of TERT. No impact of rs2853669 *TERT* -245 A>G polymorphism on outcome.	[102]
Total Papillary thyroid cancer		247/1848 (13.4%)	21/229 (9.2%)	207/229 (90.4%)					
Follicular Thyroid Cancer		13.9% 11/79	18.2% 2/11	81.8% 9/11	N/A	DNA sequencing	Patients	Only in malignant lesions.	[108]
Follicular Thyroid Cancer		66.7% 2/3	50% 1/2	50% 1/2	N/A	DNA sequencing	Patients (Korea)	No *TERTp* mutation found in 192 well-differentiated cancers without distanst metastasis.	[105]
Follicular thyroid Cancer		14% 9/64	22.2% 2/9	77.8% 7/9	Yes	DNA sequencing, qRT-PCR, IHC	Patients (Portugal)		[52]
Follicular thyroid cancer		22% 8/36	12.5% 1/8	87.5% 7/8	N/A	DNA sequencing	Patients (Sweden)	Increased prevalence in ATC: PTC: 27% (25/332); FTC: 22% (12/70); ATC: 50% (12/36).	[98]
Follicular thyroid cancer		36.4% 8/22	12.5% 1/8	87.5% 7/8	N/A	DNA sequencing	Patients (China)	Associated with older age, larger tumor size, extrathyroid invasion, advanced clinical stage. Associated with *BRAF*-V600E mutation.	[99]
Follicular thyroid cancer	III/IV	5.9% 7/119	*	*	N/A	DNA sequencing	Patients (Korea)	Associated with *BRAF*/*RAS* mutations. Associated with tumor size, stage III-IV, recurrence, decreased OS and DFS with *BRAF*/*RAS* mutations: *RAS*/*BRAF* >*TERTp* > *RAS*/*BRAF*+*TERTp*.	[106]
Follicular thyroid cancer		14% 8/58	38.5% 3/8	62.5% 5/8	Yes	DNA sequencing, WB, and IHC	Patients (Italy)	Associated with older age and poor prognosis. Increased cytoplasmic TERT. No impact of rs2853669 *TERT* -245 A>G polymorphism on outcome.	[102]
Total Follicular thyroid cancer		53/381 (13.9%)	10/46 (21.7%)	36/46 (78.2%)					
Poorly differentiated thyroid cancer		21% 3/14	33.3 1/3	66.7 2/3	Yes	DNA sequencing, qRT-PCR, IHC	Patients (Portugal)		[52]
Poorly differentiated thyroid cancer		37.5% 3/8	0% 0/3	100% 3/3	N/A	DNA sequencing	Patients	Only in malignant lesions.	[108]
Poorly differentiated thyroid cancer		29% 2/7	50% 1/2	50% 1/2	N/A	DNA sequencing	Patients (Korea)	No *TERTp* mutation found in 192 well-differentiated cancers without distanst metastasis.	[105]
Poorly differentiated thyroid cancer		51.7% 30/58	40% 12/30	60% 18/30	N/A	DNA sequencing	Patients (US & Japan)	More prevalent in advanced cancer patients with *BRAF*/*RAS^mut^*.	[100]
Total Poorly differentiated thyroid cancer		38/87 (43.7%)	14/38 (36.8%)	24/38 (63.2%)					
Anaplastic thyroid cancer		46.3% 25/54	8% 2/25	92% 23/25	N/A	DNA sequencing	Patients	Only in malignant lesions.	[108]
Anaplastic thyroid cancer		13% 2/16	50% 1/2	50% 1/2	Yes	DNA sequencing, qRT-PCR	Patients (Portugal)		[52]
Anaplastic thyroid cancer		50% 10/20	0% 0/10	100% 10/10	N/A	DNA sequencing	Patients (US & Japan)	More prevalent in advanced cancer patients with *BRAF*/*RAS^mut^*.	[100]
Anaplastic thyroid cancer		50% 10/20	20% 2/10	80% 8/10	N/A	DNA sequencing	Patients (Sweden)	PTC: 27% (25/332); FTC: 22% (12/70); ATC: 50% (12/36).	[98]
Anaplastic thyorid cancer		33.3% 12/36	*	*	N/A	DNA sequencing	Patients (Portugal & Spain)	Associated with older age, larger tumor size, distant metastases and disease-related death in FTC. PTC: 7.5% (25/332); FTC: 17.1% (12/70); PDTC: 29% (9/31); ATC: 33.4% (12/36). PTC associated with *BRAF*-V600E mutation in 60.3% of cases.	[101]
Anaplastic thyroid cancer		38.7% 41/106	10% 4/41	90% 37/41	N/A	DNA sequencing	Patients (US & China)	Associated with older age and distal metastases. −124 C>T found in 56.3% of *BRAF*-V600E mutated cases.	[104]
Total anaplastic thyroid cancer		100/252 (39.7%)	9/88 (10.2%)	79/88 (89.7%)					
Thyroid Cancer cell lines		91.7% 11/12	27.3% 3/11	72.7% 8/11	N/A	DNA sequencing	Cell lines		[108]
Thyroid Cancer cell lines		75% 6/8	17.7% 1/6	83.3% 5/6	N/A	DNA sequencing	ATC cell lines		[98]
Liver-Hepatocellular Carcinoma (HCC)									
HCC		31.4% 11/35	18,2% 2/11	81,8% 9/11	N/A	DNA sequencing	Patients (China)		[57]
HCC		34% 15/44	33.3% 5/15	66.7% 10/15	N/A	DNA sequencing	Patients (Africa, Asia, Europe)	Higher *TERTp* mutation prevalence in African (53%) compared to non-African (24%) populations.	[97]
HCC		44.3% 27/61	3.7% 1/27	96.3% 26/27	N/A	DNA sequencing	Patients (US American)	Detected in both HBV-associated and HBV-independent HCC Frequent in HCV-associated HCC.	[77]
HCC		48.5% 65/131	3.1% 2/65	96.9% 63/65	N/A	DNA sequencing	Patients (Italy)	41% of mutations in HBV-associated HCC. 53.6% mutations in HCV-associated HCC. All heterozygous. No −57 A>C.	[95]
HCC		31% 85/275	1.1% 1/85	98.9% 84/85	Yes	DNA sequencing, IHC	Patients (China)	HBV-associated HCC. Correlated with age, not with HBV status. Found in 4/7 preneoplastic lesions (HBV-associated HCC).	[63]
HCC		65.4% 68/104	3% 2/68	97% 66/68	Yes	DNA sequencing	Patients (Japan)	Associated with older age. Associated with shorter OS and DFS. Associated with HCV infection and excluded from HBV+ HCC.	[122]
HCC		58.6% 179/305	6.1% 11/179	92.7% 166/179	Yes 2–10-fold	DNA sequencing, qRT-PCR	Patients (French)	Detected in cirrhotic preneoplastic macronodules (25%) and cirrhotic adenomas (44%), at last step of malignant transformation into HCC. Absent from HBV-associated tumors 2/179 (1%) −146 C>T.	[62]
HCC		29.3% 57/195	5.3% 3/57	94.7% 54/57	No	DNA sequencing, qRT-PCR		Associated with older age. No impact on overall survival. Excluded from HBV-associated HCC. Higher frequency in HCV-associated HCC.	[96]
HCC		54% 254/469	4.3% 11/254	93% 236/254	N/A	DNA sequencing	Patients (Japan, US-European ancestry)	−57 A>C mutation detected in 1.6%. Present in 37% HBV-associated HCC but mutually exclusive with HBV sequence integration. Mutually exclusive with *TERT* CNV and *ATRX* mutations. Associated with HCV infection (64% or *TERTp* mutations). Associated with Wnt pathway mutations.	[121]
HCC		60% 9/15	11.1% 1/9	88.9% 8/9	N/A	DNA sequencing	Cell lines		[97]
Total HCC		770/1634 (47.1%)	39/770 (5%)	722/770 (93.7%)					
Cervical									
Cervical SCC		21.8% 22/101	31.8% 7/22	45.5% 10/22	Yes	qRT-PCR	Patients (Italian women)		[37]
Cervical SCC		21.4% 30/140	26.7% 8/30	73.3% 22/30	N/A	DNA sequencing, Association with clinical status	Patients (Indian women)	75% *TERTp* mutations in HPV-negative samples. −124 C>T 6/22 were TT homozygous. −146 C>T 2/8 were TT homozygous.	[36]
Cervical SCC		4.5% 1/22	100% 1/1	0% 0/1	N/A	DNA sequencing	Patients (US American)	1 patient with −125 C>A mutation.	
Total Cervical SCC		53/263 (20.1%)	16/53 (30.2%)	32/53 (60.4%)					[77]
Head and Neck Squamous Cell Carcinoma (HNSCC)									
HNSCC		31.7% 13/41	30.8% 4/13	69.2% 9/13	N/A	DNA sequencing	Patients (Indian women)	Association with clinical status.	[36]
HNSCC		17% 12/70	16.7% 2/12	83.3% 10/12	N/A	DNA sequencing	Patients (US American)	11/12 HNSCC with *TERTp* mutations were in the oral tongue, and 11/23 (47.8%) of HNSCC of the oral tongue harbored *TERTp* mutations.	[77]
Total HNSCC		25/111 (22.5%)	6/25 (24%)	19/25 (76%)					
Ovarian cancer									
Ovarian clear cell carcinoma		15% 3/20	0% 0/3	10% 2/3	N/A	DNA sequencing	Patients (US American)	1 patient with −124 C>A mutation.	[77]
Ovarian clear cell carcinoma		16.5% 37/233	8.1% 3/37	91.9% 34/37	N/A	DNA sequencing, IHC, telomere length evaluation	Patients	No link with survival or age. *TERTp* mutations tended to be mutually exclusive with loss of ARID1A protein expression and PIK3CA mutation.	[132]
Ovarian clear cell carcinoma		30% 3/10	0% 0/3	100% 3/3	Yes	qRT-PCR	Cell lines		[132]
Total ovarian clear cell carcinoma		43/263 (16.3%)	3/43 (6.9%)	39/43 (90.7%)					

N/A: not assessed; *: data not available. TERT: telomerase reverse transcriptase; GBM: glioblastoma multiforme; SCC: squamous cell carcinoma; HNSCC: head and neck squamous cell carcinoma; HCC: hepatocellular carcinoma; GI: gastrointestinal; UC: urothelial cancer; MPC: micropapillary carcinoma; HPV: Human papilloma virus; HBV: Hepatitis B virus; HCV: Hepatitis C virus; PTC: papillary thyroid cancer; FTC: follicular thyroid cancer; ATC: anaplastic thyroid cancer.

## Abbreviations

ALT	Alternative lengthening of telomeres
ATC	Anaplastic thyroid carcinoma
ATRX	α-Thalassemia/mental retardation syndrome X-linked
BCC	Basal cell carcinoma
CNS	Central nervous system
CNV	Copy number variant
DAXX	Death-domain-associated protein
DFS	Disease-free survival
DTC	Differentiated thyroid carcinoma
EGFR	Epidermal growth factor receptor
FTC	Follicular thyroid carcinoma
GBM	Glioblastoma multiforme
HBV	Hepatitis B virus
HBx	Hepatitis B X protein
HCC	Hepatocellular carcinoma
HCV	Hepatitis C virus
HNSCC	Head and neck squamous cell carcinoma
HPV	Human papillomavirus
IDH	Isocytrate dehydrogenase
OS	Overall survival
PDTC	Poorly differentiated thyroid carcinoma
PTC	Papillary thyroid carcinoma
ROS	Reactive oxygen species
SCC	Squamous cell carcinoma
TERT	Telomerase reverse transcriptase
TERTp	TERT promoter
TF	Transcription factor
TMZ	Temozolomide
TSS	Translational start site
